# Clinical characteristics and treatment strategies for pituitary adenoma associated with intracranial aneurysm

**DOI:** 10.1186/s41016-024-00370-7

**Published:** 2024-06-04

**Authors:** Zheng Huang, Zeng Yang, Lixin Xu, Haibin Leng, Kui Yang, Wei Ding, Bo Xie, Fenghua Chen, Zhixiong Liu, Zhenyan Li

**Affiliations:** 1 Department of Neurosurgery, Xiangya Hospital, Central South University, Changsha, 410008 China; 2 Department of Neurosurgery, The First People’s Hospital of Changde City, Changde, 415003 China; 3 National Clinical Research Center for Geriatric Disorders, Xiangya Hospital, Central South University, Changsha, 410008 China; 4 Research Center for Cerebrovascular Disease, Central South University, Changsha, 410008 China

**Keywords:** Pituitary adenoma; Intracranial aneurysm; Treatment strategy

## Abstract

**Background:**

This study aimed to investigate clinical features and treatment strategies for intracranial aneurysm (IA) associated with pituitary adenoma (PA).

**Methods:**

We enrolled patients with lesions in the sellar region and age-matched general population who were confirmed with IA from two hospitals. Four types of treatment strategies were performed, which included Type I (both IA and PA were treated with surgery), Type II (IA was treated with surgery and PA was performed by non-surgical treatment), Type III (PA was performed with surgery and observation was available for IA) and Type IV (both IA and PA were performed with non-surgical treatment).

**Results:**

The incidence of IA was 2.2% in the general population, 6.1% in patients with PA, 4.3% in patients with Rathke cleft cyst, 2.8% in patients with meningioma and none were found with IA in patients with craniopharyngioma. Age over 50 years (OR, 2.69; 95% CI, 1.20–6.04; *P* = 0.016), female (OR, 3.83, *P* = 0.003), and invasive tumor (OR, 3.26, *P* = 0.003) were associated with a higher incidence of IA in patients with PA. During the mean follow-up of 49.2 months, no patients experienced stroke, and recurrence of aneurysms and aneurysms treated with observation were stable. Of four patients with recurrence of PA, three patients were treated for type I and one patient for type III.

**Conclusions:**

Preoperative evaluation for aneurysm screening is necessary due to the high incidence of IA in PA patients. Our current treatment strategies may provide a benefit for these patients.

**Supplementary Information:**

The online version contains supplementary material available at 10.1186/s41016-024-00370-7.

## Background

The incidence of intracranial aneurysm (IA) in patients with pituitary adenoma (PA) has been reported as 2.3%–8.3% [1–6], which was higher than that in the general population. Unruptured IAs are more accidentally detected by preoperative radiologic evaluation for tumors, such as computed tomography angiography (CTA), which has been used as an initial diagnostic modality for aneurysm screening [7]. However, a clear association between PA and IA is still uncertain.

Although only a proportion of unruptured IA in the general population have the risk of rupture [8, 9], it remains unclear whether aneurysms in PA patients are prone to rupture so that preventive intervention could be considered. In addition, the rupture of aneurysm can be catastrophic during tumor surgery [10, 11]. Thus, the determination of management for these patients needs to weigh the risks of rupture and the risks associated with tumor surgery. Surgical clipping and endovascular treatment are effective and safe for aneurysms in the general population [7, 12, 13], and transsphenoidal endonasal surgery is the preferred approach for the vast majority of patients with PA [14–16]. However, few studies have investigated treatment strategies for PA associated with IA.

We collected the clinical and radiological data of PA patients, patients with other lesions in the sellar region, and the general population who were confirmed with IA from two hospitals in China to present the clinical features of IA in PA patients and discuss the best treatment strategies.

## Materials and methods

### Study design

The study protocol was approved by the Institutional Review Board of Xiangya Hospital. Written informed consent is obtained from all participants and/or their guardians at admission. We retrospectively reviewed patients with PA between January 2013 and June 2016 in two hospitals (Xiangya Hospital and The First People’s Hospital of Changde City) with the inclusion criteria as follows: (1) pituitary adenoma was diagnosis based on pathology and (2) intracranial aneurysm was confirmed by CTA or DSA.

In addition, to investigate the clinical features of the general population and patients with tumors in the sellar region who were associated with IA, we reviewed CTA or DSA data in the following population at the same period as the control group: (1) age-matched general population who were evaluated for general health care; and (2) patients with tumor in the sellar region.

### Neuroradiological evaluation

All patients with PA were performed with MRI and CTA before surgery. Types of tumor size were classified as microadenoma (< 10 mm), macroadenoma (10 to 40 mm), and giant adenoma (≥ 40 mm) according to maximal tumor diameter in the coronal plane of MRI.

Based on clinical manifestations, preoperative hormone assessment, and postoperative immunohistochemical analysis, patients were classified into nonfunctioning and functioning PA. The existence of invasion was confirmed by preoperative MRI according to the Knosp classification and Hardy-Wilson classification. Invasive PA included patients with Knosp III-IV or Hardy-Wilson III-IV [17, 18].

### Treatments

The treatments for PA included observations, medicine, surgical treatments, and Gamma Knife radiosurgery (Supplementary Figure S1). The surgical approach for PA included endoscopic endonasal transsphenoidal surgery and transcranial surgery. The surgical indications for PA were as follows: (1) mass effect upon surrounding structures; (2) hypopituitarism; and (3) patients presenting with hormonal dysfunction, such as acromegaly or Cushing’s disease. Transsphenoidal endoscopic endonasal surgery was the preferred approach unless patients have the following conditions: (1) parasellar tumor that extends far laterally beyond the internal carotid artery, projects anteriorly onto the planum sphenoidale, or projects laterally into the middle fossa is inaccessible from the transsphenoidal approach; (2) tumor is fibrous and adhere firmly to critical structures which is difficult to remove totally via the transsphenoidal approach and (3) prolonged sphenoid sinus inflammation. Dopaminergic drugs were preferentially chosen for the prolactinoma. Gamma Knife radiosurgery was used for residual tumors or recurrent tumors.

The treatments for IA were classified as observations and surgical treatments which included surgical clipping and endovascular intervention. Surgical decision-making for IA is based on its size, location, and morphological characteristics. The aneurysm was preferentially treated by surgery [19–21]: (1) prior hemorrhage due to rupture of IA; (2) aneurysm was over 7 mm in diameter; (3) aneurysm located in the posterior circulation;(4) multiple lobes or presence of a daughter sac; and (5) progressive development.

There were four types of treatment strategies in our institute. Type I, both IA and PA were performed with surgical treatment. Type II, IA was treated with surgery and PA was treated by observations, medicine, or Gamma Knife radiosurgery. Type III, PA requires surgical treatment and observation is available for IA. Type IV, both IA and PA were performed with non-surgical treatment (observation for IA and observation, medicine or Gamma Knife radiosurgery for PA). Patients’ choices would also be taken into consideration when the treatment strategies were applied.

### Clinical follow-up

Patients were clinically evaluated at admission, within 30 days after surgery, 3 months, and last follow-up (September 2019 or death). After discharge, the patients were followed up in the outpatient clinic and at home by telephone or visit. All outcomes were evaluated by a trained physician not directly involved in the care of these patients and blinded to the patient’s clinical data. The primary outcome was the occurrence of (1) all deaths and (2) the recurrence of IA or PA.

### Statistical analysis

Data were analyzed using IBM SPSS Statistics software (Version 20.0). Statistical significance was set at *P* < 0.05. Values were shown in the form of mean ± standard deviation (SD) for normally distributed data or median for data that were skewedly distributed. Fisher's exact test or Pearson *χ*2 test was used for the comparison of categorical variables, and quantitative variables were compared using independent Student’s *t* test, analysis of variance, or Kruskal–Wallis test. Predictors for high incidence of aneurysm in patients with PA were analyzed using a logistic regression model.

## Results

### Clinical characteristics

In total, 401 general populations, 475 patients with PA, 36 patients with meningioma of sellar region, 23 patients with Rathke cleft cyst, and 36 patients with craniopharyngioma were enrolled (Supplementary Table S1). The incidence of IA was 2.2% (9 patients) in the age-matched general population, 6.1% (29 patients) in patients with PA, 4.3% (one patient) in patients with Rathke cleft cyst, 2.8% (one patient) in patients with meningioma and no patients were found with IA in patients with craniopharyngioma.

Demographic and radiological baselines in 475 patients with PA are shown in Table 1. The mean age was 47.8 ± 11.9 years in all patients, 52.7 ± 10.0 years in patients with IA, and 47.4 ± 11.9 years in patients without IA. There were 245(51.6%) female and 230 (48.4%) male in all patients, 22 (75.9%) female and 7 (24.1%) male in patients with IA, and 223 (50.0%) female and 223 (50.0%) male in patients without IA. There were 26 (5.5%) microadenomas, 385 (81.1%) macroadenomas, and 64 (13.5%) giant adenomas in all patients. Invasive PA was found in 28.0% (133/475) of patients, and functional PA was found in 32.0% (152/475) of patients.

**Table 1:** Clinical features of pituitary adenoma associated with intracranial aneurysm

Variables	Pituitary adenoma with aneurysm (*n* = 29)	Pituitary adenoma without aneurysm (*n* = 446)	*P* value
Age(mean ± SD, years)	52.7 ± 10.0	47.4 ± 11.9	**0.021**
≥ 50 years	19(65.5%)	194(43.5%)	**0.021**
< 50 years	10(34.5%)	252(56.5%)	
Sex			**0.007**
Female	22(75.9%)	223(50.0%)	
Male	7(24.1%)	223(50.0%)	
Invasion			**0.003**
Yes	15(51.7%)	118(26.5%)	
No	14(48.3%)	328(73.5%)	
Tumor size			0.053
Microadenoma	0	26(5.8%)	
Macroadenoma	21(72.4%)	364(81.6%)	
Giant adenoma	8(27.6%)	56(12.6%)	
Hormone type			0.303
Functional	7(24.1%)	149(33.4%)	
Nonfunctional	22(75.9%)	297(66.6%)	

The entries in boldface indicates *P*<0.05, which reflects statistical significance

Of 29 PA patients with unruptured IA, there were 38 aneurysms including 21 patients with single aneurysms and 8 with multiple aneurysms (baseline characteristics were shown in Supplementary Tables S2). Twenty-one aneurysms were located in the internal carotid artery, 3 aneurysms were in the anterior cerebral artery, 1 aneurysm was in the middle cerebral artery, 7 aneurysms were in the anterior communicating artery, 6 aneurysms were in the posterior communicating artery.

## Factors associated with higher incidence of *IA* in patients with PA

The characteristics of PA patients with IA were compared with patients without IA, and results showed age (*P* = 0.021), sex (*P* = 0.007), and invasive PA (*P* = 0.003) were significantly correlated with the incidence of IA in PA patients (Table 1). We performed multivariate logistic regression analyses to define independent factors on the prevalence of IAs. Age over 50 years (OR, 2.69; 95% CI, 1.20–6.04; *P* = 0.016), female (OR, 3.83; 95% CI, 1.57–9.34; *P* = 0.003) and invasive tumor (OR, 3.26; 95% CI, 1.50–7.11; *P* = 0.003) were associated with a higher incidence of IA (Table 2).

**Table 2:** Factors for incidence of intracranial aneurysm in patients with pituitary adenoma

Characteristics	Univariate logistic regression			Multivariate logistic regression		
	OR	95%CI	*P* value	OR	95%CI	*P* value
Age (over 50 years old)	2.51	1.09–5.75	**0.030**	2.69	1.20–6.04	**0.016**
Sex (female)	3.93	1.61–9.60	**0.003**	3.83	1.57–9.34	**0.003**
Invasive pituitary adenoma	2.74	1.03–7.31	**0.044**	3.26	1.50–7.11	**0.003**
Tumor size			0.917			
Giant adenoma	1	–	–	–	–	–
Macroadenoma	0.79	0.26–2.41	0.677	–	–	–
Microadenoma	0	0–0	0.998	–	–	–
Functional pituitary adenoma	0.67	0.27–1.68	0.393	–	–	–

The entries in boldface indicates *P*<0.05, which reflects statistical significance

### Outcomes

Of 13 patients for type I, 4 patients performed interventional treatment for IA and transsphenoidal surgery for the tumor, one patient with interventional treatment and transcranial surgery for the tumor, and 8 patients with transcranial surgery for IA and PA simultaneously. Of 2 patients for type II, IA was treated with interventional surgery. Of 8 patients for type III, 5 patients with PA performed with transsphenoidal surgery, and 3 patients with transcranial surgery. Of 6 patients for type IV, one patient was treated by medicine, one patient was treated by Gamma Knife radiosurgery, and four patients were treated with observation (Table 3).

**Table 3:** Clinical outcome of pituitary adenoma patients with intracranial aneurysm

	No. of patients	Perioperative complications	Recurrence of aneurysm	Recurrence of pituitary adenoma	Death
Type I	13	1	0	3	0
Type II	2	0	0	0	0
Type III	8	0	0	1	0
Type IV	6	0	0	0	1

Knife radiosurgery and observation are applied for aneurysm

*Type I* surgical treatment for aneurysm and tumor, *Type II* aneurysm is treated with surgery and tumor is treated by observations, medicine, or Gamma Knife radiosurgery, *Type III* tumor is treated with surgery and observation is available for aneurysm, *Type IV* tumor is treated by observations, medicine or Gamma

In type I, diabetes insipidus occurred in one patient and seizure in one patient within 30 days after surgery. During the mean follow-up of 49.2 months (range, 10–80 months), no patients experienced stroke or recurrence of IA. Aneurysms treated with observation were stable. Of four patients with recurrence of PA, three patients were treated for type I, and one patient was treated for type III. The overall mortality rate was 3.4% and one patient died 2 years after surgery due to lung cancer.

## Discussion

The co-occurrence of IA and PA was first described by Housepian and Pool in 1958 [1], and several studies subsequently reported the incidence was 2.3–8.3% [2–6], which was consistent with our results. In addition, the incidence of aneurysm was higher in PA patients (6.1%) than in the general population (2.2%) and other lesions in the sellar region (none in the craniopharyngioma, 2.8% in the meningioma and 4.3% in the Rathke cleft cyst). Preoperative angiography is not commonly used in patients with intracranial tumors, except for assessing blood supply for tumors. Our results demonstrated preoperative evaluation for screening aneurysms was necessary due to the high incidence of aneurysms in PA patients. Although DSA remains the golden standard for the diagnosis of aneurysm [22], CTA is a more rapid, less invasive, and more cost-effective procedure compared with DSA [23]. Besides, the high sensitivity and specialty of CTA for initial detection of aneurysm has been confirmed [7, 24]. Moreover, CT is very useful in identifying mural calcification and thrombus which can have a significant impact on treatment decisions for IA, and the sphenoid septal anatomy is better visualized on high-resolution CT for preoperative assessment of PA [25, 26]. Thus, CTA added to high-resolution CT was used as a regular examination for skull base tumors in our institute. If the aneurysm is not confirmed by CTA, DSA is further recommended. Thus, preoperative CTA may be recommended for screening aneurysm in patients with PA.

The factors that contribute to the formation of IA in patients with PA remain unclear and there are some factors reported in previous studies that might explain the high association between PA and IA, which are in accordance with our results. Our study found that tumor invasion was associated with a high incidence of IA, and the possible mechanism may be as follows. On the one hand, direct mass effect and invasion of a brain tumor may influence the vascular structure and hemodynamics of major cerebral arteries in the skull base [3, 27, 28]. On the other hand, abnormal hormone secretion and other biochemical factors have been supposed to be another factor [3, 29, 30]. Jakubowski and Kendall reviewing 150 patients found that IA was more commonly found in growth hormone (GH)-secreting PA than in nonfunctional PA (13.8% vs. 5.1%) [3]. An elevated level of GH and insulin-like growth factor-I induces arteriosclerosis and degenerative changes in the major cerebral artery of the skull base which further promote aneurysm formation [5]. In our cohort, we did not find an increased occurrence of aneurysms in GH-secreting tumors (5.1% vs. 6.9%) or other functional tumors, which may explain why most patients with GH-secreting tumors were early diagnosed and then timely treated by surgery before the formation of aneurysm. Although the underlying reasons for the association between PA and IA require further studies, our study revealed that older PA patients (age over 50 years), female patients, and patients with invasive tumors, should be more careful with preoperative screening aneurysms.

Treatment strategies for PA associated with IA need to weigh the risks of rupture and benefits associated with tumor surgery to establish the best management. Both PA and IA need to be surgically treated and two options were available. Aneurysm clipping and tumor resection could be performed simultaneously via the craniotomy approach, depending on the following conditions. First, progressive symptoms related to tumors need to be treated as soon as possible, such as pituitary apoplexy [31]. Additionally, a high risk of rupture is identified by patients’ characteristics and morphologic features of aneurysm, which is inappropriate for treatment after tumor surgery [32]. Second, tumor characteristics are inaccessible from the transsphenoidal approach. Of 8 patients treated for this management strategy in our study, one patient experienced a recurrence of the tumor and none was found recurrence of aneurysm in the follow-up. In general, this strategy is optional in some conditions.

Another option is staged surgery. Endoscopic endonasal transsphenoidal surgery is the preferred approach in the majority of patients with PA unless the patient’s condition or tumor characteristics are not suitable for this approach [14, 15, 33–35]. An aneurysm should be treated before tumor surgery depending on its location and morphologic features [36] because the ICA may be inadvertently injured and the aneurysm experience rupture during tumor surgery[10, 11, 37]. Endovascular intervention of aneurysm is less invasive and thus more commonly used. The use of antiplatelet drugs in patients with stents would influence tumor surgery. In type I, all 5 patients with staged surgery were chosen for endovascular treatment for an aneurysm and there was no recurrence of an aneurysm in the follow-up. The tumor in these patients was growing slowly without urgent clinical symptoms, and a delay in tumor surgery did not matter. According to previously reported cases and our results, staged surgery is also an effective and safe treatment.

## Limitations

This study has several limitations. First, the mechanism of the co-occurrence of PA and IA will help identify high-risk patients that preventive management can be applied, and it should be explored completely in future study. Second, it was a retrospective study conducted in two hospitals, and the condition of PA patients with aneurysms is more complicated. Our treatment strategies need to be further investigated through multicenter and large-sample studies.

## Conclusions

The incidence of IA is higher in the patients with PA than in the age-matched general population and other lesions in the sellar region. Age over 50 years, female, and invasive PA were predictors for increased incidence of IA. Preoperative evaluation for screening aneurysms is necessary in these patients. Our current treatment strategies may provide a benefit for PA patients with IA. Multi-center prospective studies should be further performed.

### Supplementary Information

Additional file 1: Supplementary Table S1. Clinical characteristics in general populations and patients with lesions in the sellar region. Supplementary Table S2. Clinical characteristics in pituitary adenoma patients with intracranial aneurysm. Supplementary Figure S1. Patients who underwent interventional treatment for aneurysm and Gamma knife for tumor.

## Data Availability

The datasets used and/or analyzed during the current study are available from the corresponding author on reasonable request.

## Abbreviations

CTA: Computed tomography angiography
DSA: Digital subtract angiography
GH: Growth hormone
IA: Intracranial aneurysm
MRI: Magnetic resonance imaging
PA: Pituitary adenoma
