# Supplementary material for: Clinical characteristics and treatment strategies for pituitary adenoma associated with intracranial aneurysm
Source: Chin Neurosurg J. 2024 Jun 4;10:18. doi: 10.1186/s41016-024-00370-7 (PMC11149326; doi:10.1186/s41016-024-00370-7)
Supplement: Supplementary file 1 — Additional file 1: Supplementary Table S1. Clinical characteristics in general populations and patients with lesions in the sellar region. Supplementary Table S2. Clinical characteristics in pituitary adenoma patients with intracranial aneurysm. Supplementary Figure S1. Patients who underwent interventional treatment for aneurysm and Gamma knife for tumor. [file 41016_2024_370_MOESM1_ESM.docx]

**Supplementary material**

**Table S1. Clinical characteristics in general populations and patients with lesions in the sellar region**

|  | General population | Patients with Pituitary adenoma | Patients with Rathke cleft cyst | Patients with meningioma | Patients with craniopharyngioma |
| --- | --- | --- | --- | --- | --- |
| No. of patients | 401 | 475 | 23 | 36 | 43 |
| Age (mean±SD, years) | 45.9±14.8 | 47.8±11.9 | 41.35±14.90 | 48.80±11.53 | 42.21±14.84 |
| Sex (Female) | 183 (45.6%) | 245 (51.6%) | 18 (78.3%) | 29 (80.6%) | 17 (39.5%) |
| Smoking | 46 (11.5%) | 57 (12.0%) | 2 (8.7%) | 5 (13.9%) | 4 (9.3%) |
| Alcohol consumption | 22 (5.5%) | 26 (5.5%) | 2 (8.7%) | 2 (5.6%) | 3 (7.0%) |
| Associated aneurysm | 9 (2.2%) | 29 (6.1%) | 1(4.3%) | 1 (2.8%) | 0 |
| No. of aneurysm | 10 | 38 | 1 | 1 | 0 |
| Location of aneurysm |  |  |  |  | - |
| Internal carotid artery | 7 | 21 | 0 | 0 | 0 |
| Anterior communicating artery | 1 | 7 | 0 | 1 | 0 |
| Posterior communicating artery | 0 | 6 | 0 | 0 | 0 |
| Anterior cerebral artery | 0 | 3 | 0 | 0 | 0 |
| Middle cerebral artery | 1 | 1 | 0 | 0 | 0 |
| Posterior cerebral artery | 0 | 0 | 1 | 0 | 0 |
| Basilar artery | 1 | 0 | 0 | 0 | 0 |

Table S2. **Clinical characteristics in pituitary adenoma patients with intracranial aneurysm**

| Treatment strategies* | No. of patients | Sex | Age | Location of aneurysm | Size of aneurysm | Shape of aneurysm (multiple lobes or presence of a daughter sac) | Aneurysm near the tumor or aneurysm inside of the tumor | Size of tumor | Type of tumor |
| --- | --- | --- | --- | --- | --- | --- | --- | --- | --- |
| Type I | Case 1 | Male | 66 | ICA | 5mm*5mm | No | Yes | 4cm*3cm*3cm | NF |
|  | Case 2 | Female | 66 | ICA | 7mm*4mm | Yes | Yes | 5cm*5cm*3cm | NF |
|  | Case 3 | Female | 50 | PcomA | 5mm*3.6mm | Yes | No | 3cm*2.6cm*2cm | NF |
|  | Case 4 | Female | 57 | PcomA | 3.75mm*3.45mm | Yes | No | 3cm*2cm*2cm | NF |
|  | Case 5 | Male | 52 | AcomA | 3.1mm*2.4mm | Yes | No | 2cm*2cm*2cm | NF |
|  | Case 6 | Female | 58 | ICA, PcomA | 2.7mm*1.8mm,2mm*1.9mm | No | Yes | 3.5cm*2.4cm*2.2cm | NF |
|  | Case 7 | Female | 63 | AcomA, ACA | 3.7mm*3.1mm,4mm*3.8m | Yes | No | 2.2cm*1.9cm*1.6cm | GH |
|  | Case 8 | Female | 63 | AcomA, ACA | 2.5mm*2.2mm，5.1mm*3.2mm | Yes | No | 4cm*4cm*3cm | NF |
|  | Case 9 | Female | 41 | ICA | 3.4mm*2.3mm | No | Yes | 2.6cm*2.4cm*1.6cm | NF |
|  | Case 10 | Female | 54 | ICA | 2.4mm*1.9mm | No | Yes | 4.7cm*2.9cm*2.5cm | NF |
|  | Case 11 | Female | 50 | ICA, PcomA | 5.2mm*5.2mm，2.6mm*2.5mm | Yes | Yes | 2.4cm*2.2cm*4cm | GH |
|  | Case 12 | Female | 48 | ICA | 9.1mm*5.7mm | Yes | Yes | 5cm*5cm*4cm | NF |
|  | Case 13 | Female | 49 | ICA | 2mm*1.8mm | Yes | Yes | 3.5mm*3.4mm*2.5m m | NF |
| Type II | Case 1 | Female | 49 | L-ICA，R-ICA | 3.85*3.83mm，6.36*4.89mm | Yes | No | 2.1cm*1.6cm*1cm | NF |
|  | Case 2 | Female | 26 | ICA | 2.4mm*1.7mm | Yes | No | 1.8cm*1cm*0.9cm | PRL |
| Type III | Case 1 | Female | 56 | ICA | 1.5mm*1.2mm | No | No | 5cm*5cm*6cm | NF |
|  | Case 2 | Female | 49 | L-ICA, L-ICA, R-ICA | 6mm*3mm，3mm*2mm，1mm*1mm | No | Yes | 5cm*5cm*4cm | NF |
|  | Case 3 | Female | 41 | PcomA | 1.8mm*1.4mm | No | No | 5cm*5cm*4cm | PRL |
|  | Case 4 | Female | 34 | ICA | 2.0mm*1.5mm | No | No | 2cm*2cm*1.5cm | PRL |
|  | Case 5 | Male | 39 | AcomA | 2.8mm*1.2mm | No | No | 2.8cm*1.8cm*1.8cm | NF |
|  | Case 6 | Female | 64 | MCA | 5.2mm*4.6mm | No | No | 1.5cm*1.5cm*1.5cm | NF |
|  | Case 7 | Male | 56 | AcomA | 7.1mm*4.3mm | No | No | 3cm*2cm*2cm | NF |
|  | Case 8 | Female | 60 | ICA | 7.9mm*5mm | No | No | 2cm*2cm*2cm | NF |
| Type IV | Case 1 | Male | 52 | ICA | 1.6mm*1.2mm | No | No | 2cm*1.8cm*1.5cm | NF |
|  | Case 2 | Male | 58 | ICA | 4mm*3mm | No | Yes | 3.3cm*2.9cm*1.7cm | PRL |
|  | Case 3 | Female | 44 | AcomA | 4mm*4mm | No | No | 2.4cm*2.3cm*2.2cm | GH |
|  | Case 4 | Female | 56 | PcomA, AcomA | 2.7mm*2.7mm，2.3mm*2.0mm | No | No | 6.1cm*4.1cm*3cm | NF |
|  | Case 5 | Male | 61 | ICA | 25.5mm*18.7mm | No | Yes | 3.3cm*3.2cm*3cm | NF |
|  | Case 6 | Female | 66 | ACA, ICA | 2.2mm*1.2mm，3.8*2.4mm， | No | No | 2.1cm*15cm*1.5cm | NF |

* Type I: Surgical treatment for aneurysm and tumor; Type II: Aneurysm is treated with surgery and tumor is treated by observations, medicine or Gamma Knife

radiosurgery; Type III: Tumor is treated with surgery and observation is available for aneurysm. Type IV: Tumor is treated by observations, medicine or Gamma

Knife radiosurgery and observation is applied for aneurysm.

ICA: internal carotid artery. AcomA: anterior communicating artery. PcomA: posterior communicating artery. ACA: anterior cerebral artery. MCA: middle cerebral artery

NF: nonfunctional. GH: growth hormone. PRL: prolactin


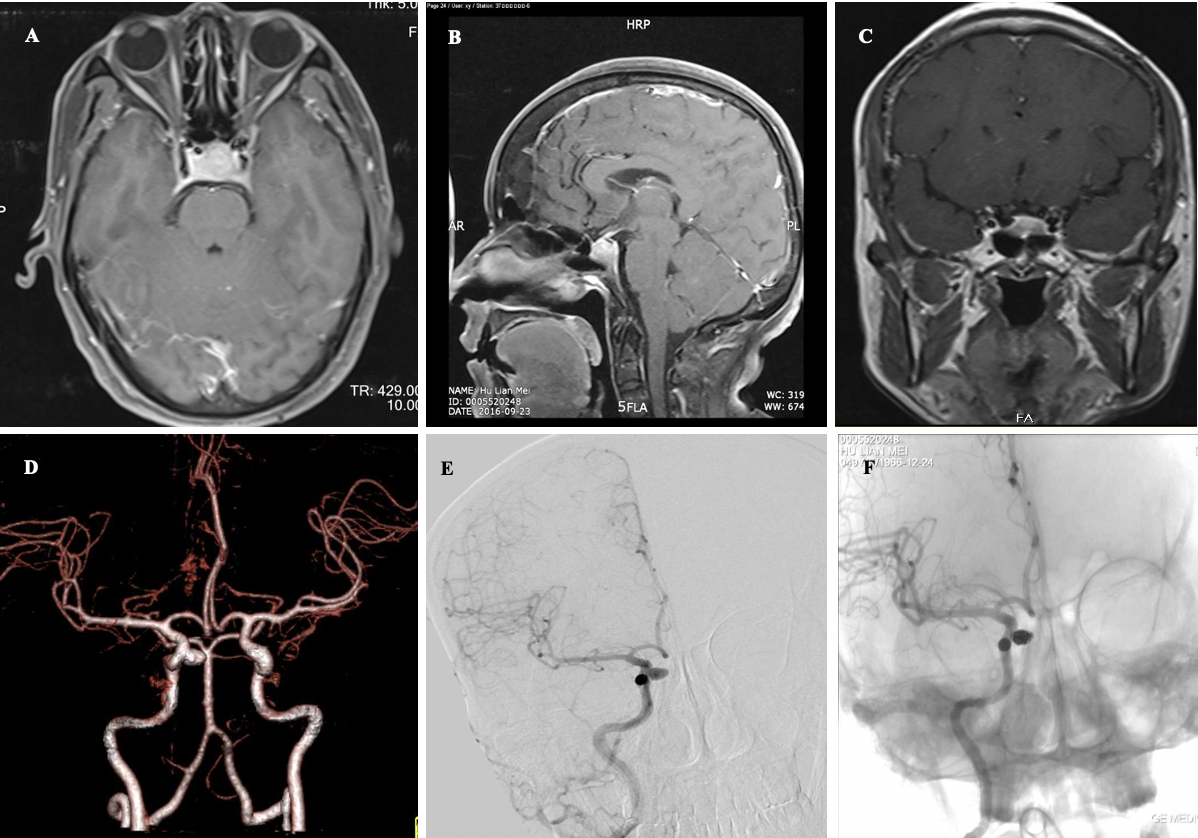


Figure S1. Patients who underwent interventional treatment for aneurysm and Gamma knife for tumor. A, B, C. MRI showed the relationship between tumor and aneurysm. D. CTA showed an aneurysm in the right ICA. E. DSA showed an aneurysm in the right ICA. F. Postoperative DSA showed the ICA aneurysm which was treated by endovascular coiling.
